# Alterations in B cell subsets correlate with body composition parameters in female adolescents with anorexia nervosa

**DOI:** 10.1038/s41598-020-80693-4

**Published:** 2021-01-13

**Authors:** Jana Freff, Kathrin Schwarte, Lisa Bröker, Judith Bühlmeier, Isabelle Kraft, Dana Öztürk, Anke Hinney, Volker Arolt, Udo Dannlowski, Georg Romer, Bernhard T. Baune, Johannes Hebebrand, Manuel Föcker, Judith Alferink

**Affiliations:** 1 Department of Mental Health, University of Münster, Albert-Schweitzer-Campus 1, 48149 Münster, Germany; 2 Cells in Motion Interfaculty Cluster, University of Münster, 48149 Münster, Germany; 3 Department of Child and Adolescent Psychiatry and Psychotherapy, University Hospital Essen, University of Duisburg-Essen, 45147 Essen, Germany; 4 Department of Child and Adolescent Psychiatry and Psychotherapy, University Hospital Münster, 48149 Münster, Germany; 5 The Florey Institute of Neuroscience and Mental Health, The University of Melbourne, Melbourne, 3010 Australia; 6 Department of Psychiatry, The University of Melbourne, Melbourne, 3010 Australia

**Keywords:** Immunology, Psychology, Diseases, Medical research

## Abstract

Anorexia nervosa (AN) is a severe eating disorder and often associated with altered humoral immune responses. However, distinct B cell maturation stages in peripheral blood in adolescents with AN have not been characterized. Treatment effects and the relationship between clinical and B cell parameters are also not fully understood. Here we investigated the phenotype of circulating B cell subsets and the relationship with body composition in adolescents with AN before (T0, n = 24) and after 6 weeks (T1, n = 20) of treatment. Using multi-parameter flow cytometry, we found increased percentages of antigen-experienced B cells and plasmablasts in patients with AN compared to healthy controls (n = 20). In contrast, percentages of CD1d^+^CD5^+^ B cells and transitional B cells with immunoregulatory roles were reduced at T0 and T1. These B cell frequencies correlated positively with fat mass, fat mass index (FMI), free fat mass index, and body mass index standard deviation score. In addition, scavenger-like receptor CD5 expression levels were downregulated on transitional B cells and correlated with fat mass and FMI in AN. Our findings that regulatory B cell subgroups were reduced in AN and their strong relationship with body composition parameters point toward an impact of immunoregulatory B cells in the pathogenesis of AN.

## Introduction

Anorexia nervosa (AN) is a serious eating disorder (ED) that is particular common in female adolescents and young women^1^. It is characterized by significantly low body weight due to food restriction, an intense fear of gaining weight, body image disturbances, and behavior interfering with weight gain. AN has the highest mortality rate of all mental illnesses due to starvation-induced complications and an elevated suicide risk^2^. Body composition is significantly altered in AN. Accordingly, lower fat mass and fat free mass were found in patients with AN associated with an extreme physiological state including numerous endocrine and metabolic adaptions^3,4^. Furthermore, hematological changes and a respectively increased risk of infection seem to be associated with chronicity of AN, weight loss and the severity of underweight^5^. Genetic and environmental factors have been shown to influence disease risk^6–8^. Accumulating evidence indicates that immune mechanisms are also involved in the pathogenesis of AN^9^. However, the underlying mechanisms are far from being understood.

Inflammatory processes are involved in both, adipose tissue deposition and fasting. Both in humans and in rodents, immune cells of the innate and adaptive immune system have been detected in adipose tissue^10^. Animal studies further indicate that inflammation impacts fat mass loss induced by fasting^11^. A plethora of studies suggested that inflammatory processes play a critical role in the pathophysiology of AN^9^. For example, a bidirectional relationship between ED and autoimmune diseases has been demonstrated^12^. In a large Swedish cohort, previous autoimmunity such as type 1 diabetes mellitus was associated with increased risk for ED and, vice versa, AN increased the subsequent risk of gastrointestinal-related autoimmune diseases^13^. Furthermore, genome-wide association studies (GWAS) demonstrated a close link between the immune system and AN^14^. A significant locus on chromosome 12 has been identified for AN, and SNP rs4622308 is in high linkage disequilibrium with a known GWAS hit for autoimmune disorders type 1 diabetes mellitus and rheumatoid arthritis^6^. In addition, elevated levels of proinflammatory cytokines have been found in peripheral blood (PB) of patients with AN. Meta-analyses revealed elevated levels of proinflammatory Tumor Necrosis Factor (TNF), Interleukin (IL)-1β and IL-6 in patients with AN compared to healthy controls pointing toward a mild proinflammatory status in AN^15,16^. Also cellular components of the innate and adaptive immune system are modulated in AN. Reduced numbers of neutrophils and NK cells have been observed and neutrophilic chemotaxis and adherence were shown to be impaired in AN^9^. Regarding the adaptive immune system, several studies found increased CD4/CD8 T cell ratios in PB due to a reduction in CD8^+^ T cell counts among overall T cells^17,18^. Controversial results have been reported regarding T cell proliferation in AN with enhanced, equivalent or even reduced responses depending on the mitogens used^9^.

B cells play a versatile role in humoral immunity, in both pro- and anti-inflammatory processes through antibody production, secretion of pro- and anti-inflammatory cytokines and chemokines, and antigen-presentation^19^. Based on surface marker expression and maturation phenotype, human CD19^+^ B cell subtypes have been classified into (i) transitional B cells (CD24^+^CD38^hi^) which represent immature, recent emigrants from the bone marrow, (ii) naïve-mature B cells (CD27^-^IgD^+^), (iii) antigen-activated switched (CD27^+^IgD^−^), and (iv) non-switched memory B cells (CD27^+^IgD^+^), (v) regulatory B cells (CD1d^+^CD5^+^), and (vi) plasmablasts (CD24^-^CD38^hi^) with the capacity to differentiate into antibody secreting plasma cells^19,20^. Several studies found B cell numbers and percentages in AN equivalent to normal controls; with the exception of a recent study that reported increased B cell counts, but equivalent percentages of these cells in adolescents with AN^17,21–23^. However, up to now, B cell subsets of diverse maturation states have not been fully studied in anorectic adolescents. There is also a lack of knowledge about the B cell compartment before and after therapy and the link to body composition parameters.

In this study, we conducted immune cell phenotyping of peripheral blood mononuclear cells (PBMC) in adolescents with AN, with a particular emphasis on B cells. We phenotypically characterized distinct B cell subpopulations including transitional, naïve-mature, antigen-experienced switched, and non-switched memory B cells, regulatory B cells, and plasmablasts by flow cytometry and studied treatment effects on the B cell compartment. We further report the results of correlational studies and linear regression analyses that determined relationships between body mass index (BMI)/body composition parameters and B cell subset percentages and markers.

## Results

Characteristics of the study sample are summarized in Table 1. For the majority of the patients it was the first inpatient admission and mean premorbid BMI parameters were in the normal range (see Supplementary Table S1). Mean age at admission (T0) did not differ significantly (*p* = 0.248) between patients (15.6 ± 1.4 years) and healthy controls (HC) (16.1 ± 1.6). Compared to HC, means of BMI, BMI percentile, and BMI SDS were significantly lower, both at inpatient admission (T0) and after 6 weeks of treatment (T1) (*p* < 0.001). At T1, BMI, BMI percentile, and BMI standard deviation score (BMI SDS) significantly increased compared to T0 (*p* < 0.001). Body composition parameters including fat mass (FM), fat mass index (FMI), and free fat mass index (FFMI) were significantly reduced at inpatient admission (T0) compared to HC (*p* < 0.001). At T1, the FFMI remained unchanged between T0 and T1. However, FM significantly increased at T1 compared to T0 (*p* < 0.001) but was still lower compared to HC (*p* < 0.05). FMI at T1 was significantly higher when compared to T0 and HC (*p* < 0.001).

**Table 1:** Characteristics of the study sample.

	HC	AN		*p*-value
	n = 20	T0, n = 24 (n = 20)*	T1, n = 20	
	Mean ± SD	Mean ± SD	Mean ± SD	
Body height (cm)	169.1 ± 6.7	165.4 ± 5.1	165.4 ± 5.4	**0.045**^**a**^ 0.057^b^ 0.066
		(165.1 ± 5.5)		
Weight (kg)	58.9 ± 7.9	43.9 ± 5.1	47.7 ± 5.1	**< 0.001**^**a,b,c**^
		(44.5 ± 4.9)		
BMI (kg/m^2^)	20.5 ± 1.9	16.0 ± 1.5	17.4 ± 1.2	**< 0.001**^**a,b,c**^
		(16.3 ± 1.2)		
BMI percentile (according to KIGGS)	39.3 ± 23.7	2.8 ± 3.4	7.4 ± 5.6	**< 0.001**^**a,b,c**^
		(2.9 ± 3.4)		
BMI SDS (according to KIGGS)	− 0.4 ± 0.7	− 2.6 ± 1.5	− 1.7 ± 0.8	**< 0.001**^**a,b,c**^
		(− 2.4 ± 1.1)		
FM (%)	25 ± 5.6^#^	17 ± 4.3^+^	21.5 ± 5	**< 0.001**^**a,c**^ **0.049**^**b**^
		(17.1 ± 4.1)		
FMI (kg/m^2^)	5.2 ± 1.5^#^	2.8 ± 0.8^+^	7.9 ± 1.8	**< 0.001**^**a,b,c**^
		(2.8 ± 0.7)		
FFMI (kg/m^2^)	15.3 ± 1.4^#^	13.4 ± 1.2^+^	13.7 ± 1.1	**< 0.001**^**a,b**^ 0.087^c^
		(13.5 ± 1.2)		

Significant effects are in bold print. *HC* healthy control, *AN* patients with Anorexia nervosa, *BMI* body mass index, *SDS* standard deviation scores, *FM* fat mass, *FMI* fat mass index, *FFMI* fat free mass index, *KIGGS* German Health Interview and Examination Survey for Children and Adolescents. *P-*values vs. HC were calculated by *t* test for independent samples, T0 vs. T1 by *t* test for paired samples.

*20 AN patients with follow-up data, ^#^n = 19, ^+^n = 23

^a^HC vs. AN(T0).

^b^HC vs. AN(T1).

^c^AN(T0) vs. AN(T1).

### Altered frequencies of total lymphocytes but not CD19^+^ B cells in adolescents with AN

**Figure 1:**
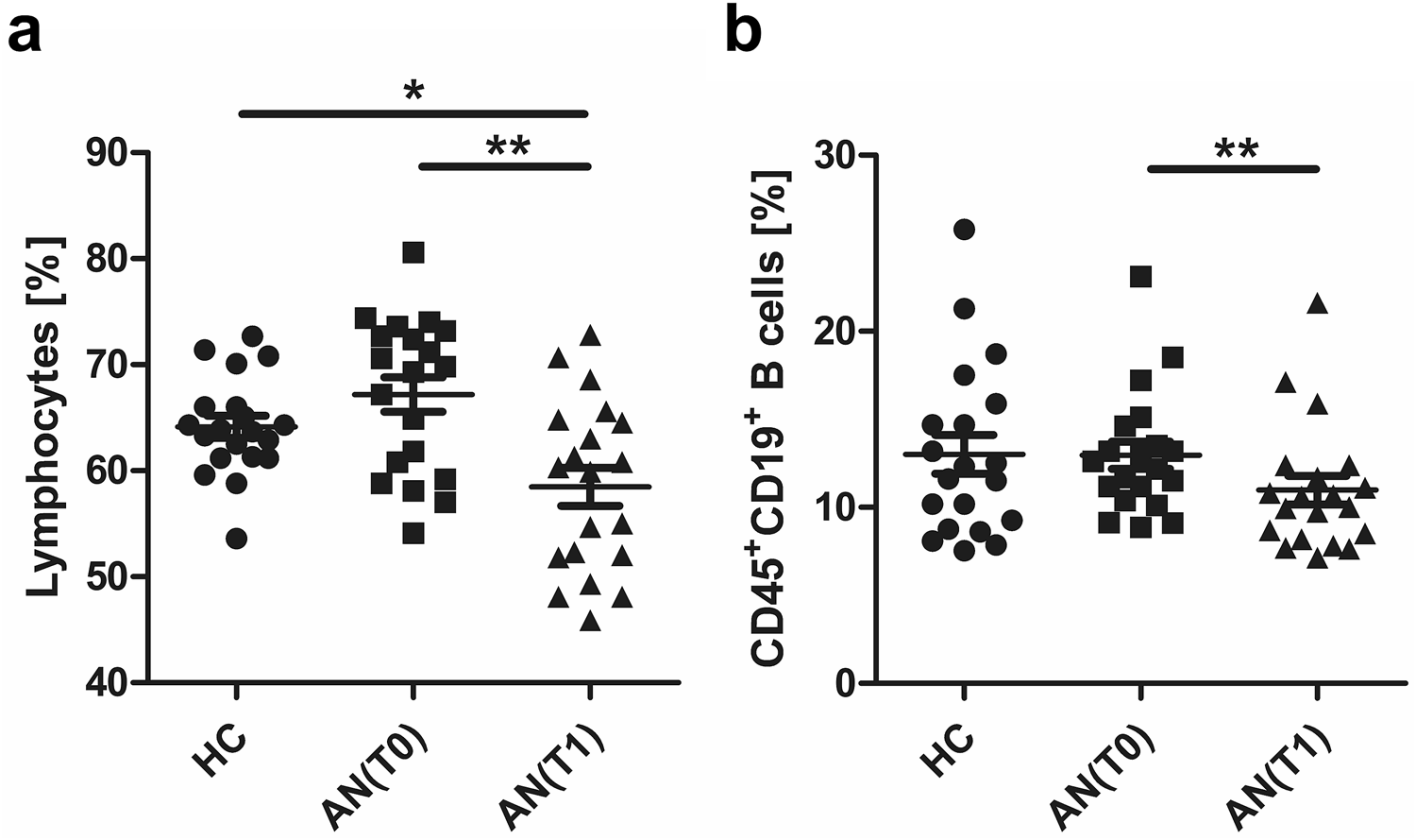
Lymphocytes and B cells in PBMC of healthy controls (HC) and adolescents with anorexia nervosa (AN). Graphs displaying frequencies of (**a**) lymphocytes and (**b**) CD45^+^CD19^+^ B cells in HC (n = 20) and AN at T0 (n = 20) and at T1 (n = 20). *P-*values vs. HC were calculated by *t* test for independent samples, T0 vs. T1 by *t* test for paired samples, *p < 0.05; **p < 0.01.

To study B cell subsets in peripheral blood mononuclear cells (PBMC) of adolescents with AN before and after 6 weeks of inpatient treatment, we first assessed percentages of total lymphocytes and CD19^+^ B cells using multi-parameter flow cytometry. We found equivalent frequencies of total lymphocytes among PBMC in patients with AN at T0 when compared to HC. At T1, proportions of total lymphocytes were reduced in AN when compared to T0 and HC (Fig. 1a). Frequencies of circulating CD19^+^ B cells were also reduced at T1 compared to T0 (Fig. 1b).

### Reduced frequencies of IgD^+^CD27^−^ naïve B cells and increased IgD^−^CD27^+^ switched memory B cell percentages in adolescents with AN

**Figure 2:**
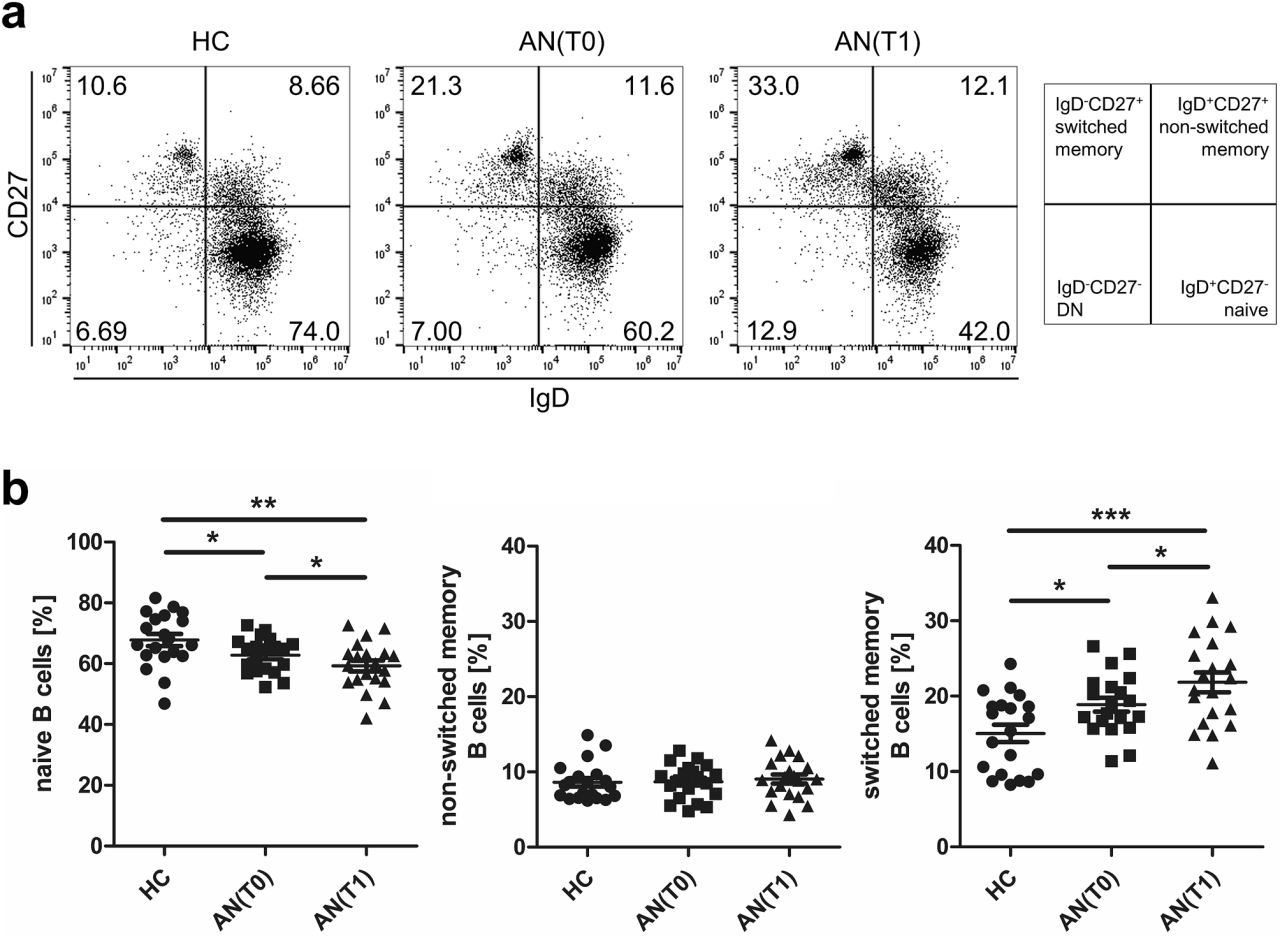
Naïve and memory B cells in PBMC of healthy controls (HC) and adolescents with anorexia nervosa (AN). (**a**) Representative dot plots of HC, patients with AN at T0 and T1 showing IgD^+^CD27^−^ naive, IgD^+^CD27^+^ non switched memory, IgD^−^CD27^+^ switched memory and IgD^−^CD27^−^ DN B cell populations, pre-gated on CD45^+^CD19^+^ B cells in PB lymphocytes. (**b**) Graphs displaying frequencies of naïve, non-switched memory and switched memory B cells in HC (n = 20) and patients with AN at T0 (n = 20) and at T1 (n = 20). *P-*values vs. HC were calculated by *t* test for independent samples, T0 vs. T1 by *t* test for paired samples, *p < 0.05; **p < 0.01; ***p < 0.001.

To characterize circulating B cell subsets in adolescents with AN in more detail, we used an earlier established classification of B cell subpopulations according to their maturation stages^24^. We assessed transitional, naïve-mature, antigen-experienced and non-switched memory cells, plasmablasts, and regulatory B cells by surface marker expression of CD19, CD1d, CD5, CD24, CD27, CD38, and IgD by flow cytometry. We first examined naïve and memory B cells in patients and HC by assessment of IgD and CD27 co-expressing CD19^+^ B cells (Fig. 2a). Naïve (IgD^+^CD27^−^) B cells were decreased at T0 compared to HC and even decreased further after 6 weeks of treatment (Fig. 2b). Equivalent percentages of non-switched memory (IgD^+^CD27^+^) B cells were found in PB of adolescents with AN at T0 when compared to HC and T1 (Fig. 2b). Conversely, switched memory (IgD^−^CD27^+^) B cell proportions increased markedly in comparison to the equivalent cell type in PB of HC (Fig. 2b). At T1, frequencies of this B cell subset even further increased. In sum, these findings demonstrate an increase in percentages of antigen-experienced B cells and plasmablasts in AN, both functionally involved in antibody-mediated immune responses^25^.

**Figure 3:**
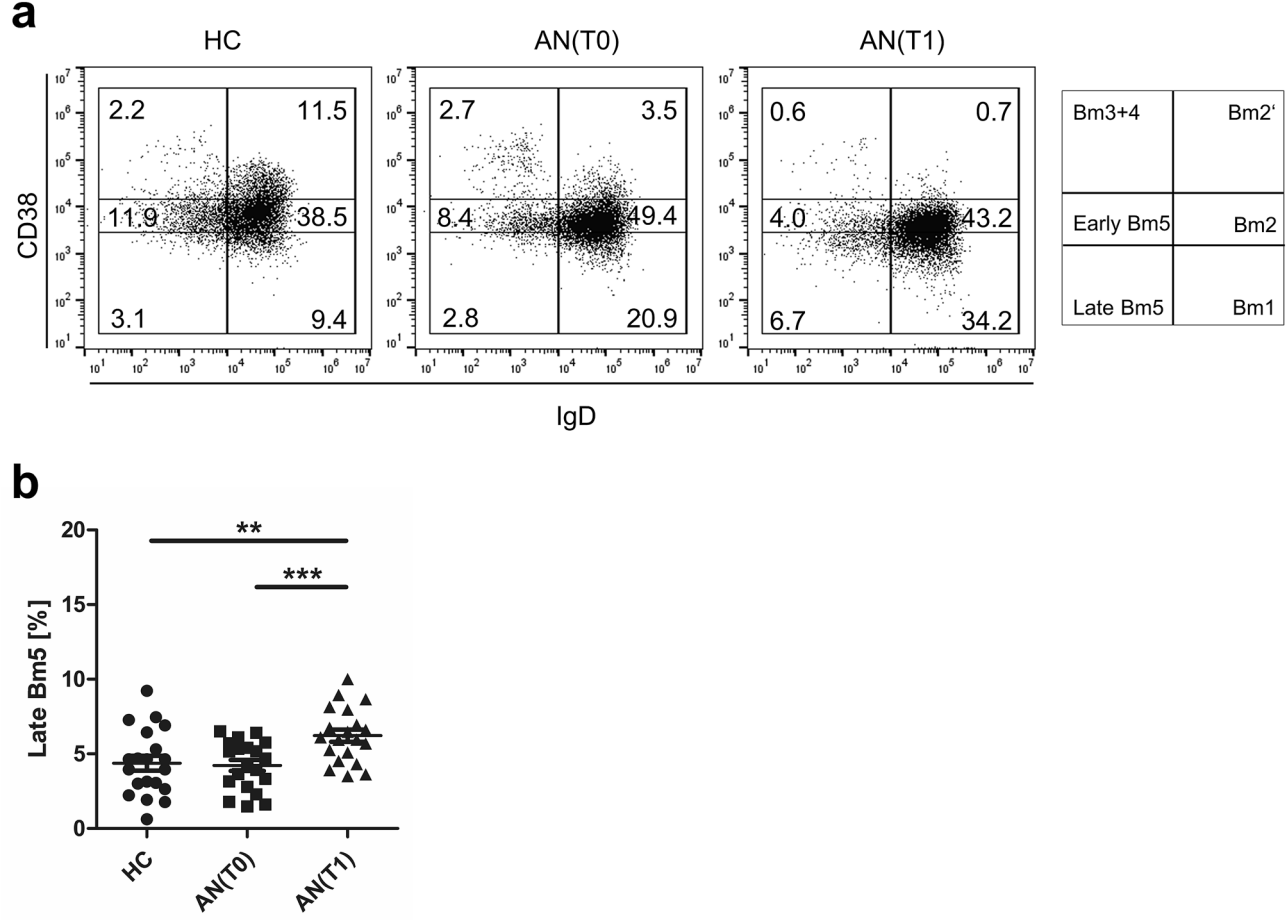
Late Bm5 cells in PBMC of healthy controls (HC) and adolescents with anorexia nervosa (AN). (**a**) Representative dot plots of HC, patients with AN at T0 and T1 showing Bm1-5 classification based on surface expression of CD38 and IgD, pre-gated on CD45^+^CD19^+^ B cells in PB lymphocytes. (**b**) Graph displaying the frequencies of late Bm5 cells (CD38^−^IgD^−^) in HC (n = 20) and in patients with AN at T0 (n = 20) and at T1 (n = 20). *P-*values vs. HC were calculated by *t* test for independent samples, T0 vs. T1 by *t* test for paired samples, **p < 0.01; ***p < 0.001.

We next assessed IgD and CD38 surface expression on B cells to differentiate them according to the Bm1–Bm5 classification^26^. In analogy to human tonsillar Bm subpopulations, five mature B cell (Bm) subpopulations have been identified before in human PB^27^. We found that Bm1–Bm4 cells were unchanged in the study groups. Frequencies of Bm2′ cells decreased at T0 in patients with AN compared to HC and even further decreased at T1. However, frequencies of early memory (Bm5) cells increased at T1 compared to HC (Fig. 3a, and see Supplementary Fig. S1). In addition, frequencies of late Bm5 cells that constitute late memory B cells, increased in AN patients at T1 when compared to patients at admission and HC (Fig. 3b).

### Altered frequencies of plasmablasts and regulatory B cells in adolescents with AN

**Figure 4:**
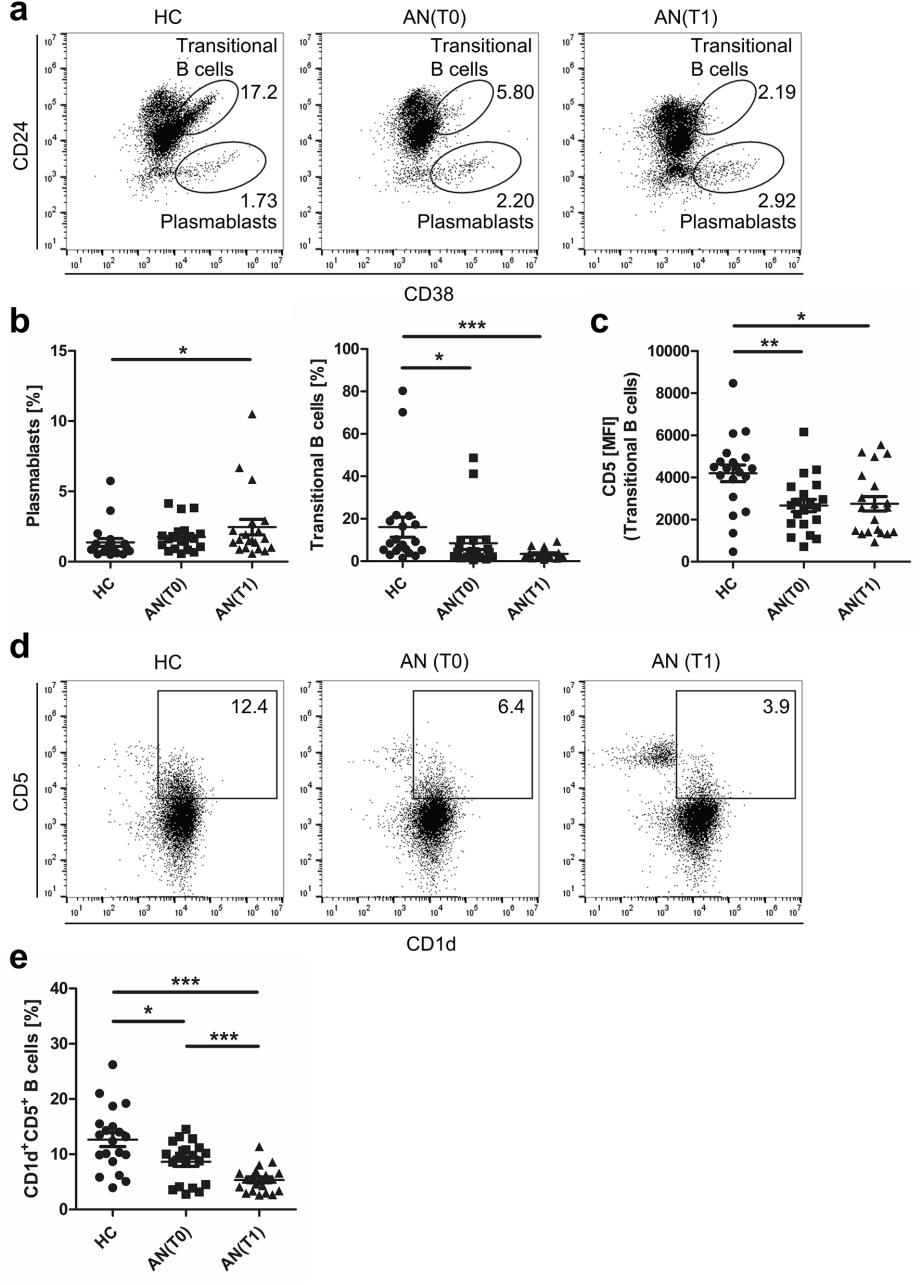
Plasmablasts and transitional B cells and CD1d^+^CD5^+^ B cells in PBMC of healthy controls (HC) and adolescents with anorexia nervosa (AN). (**a**) Representative dot plots of a HC and patients with AN showing CD24^+^CD38^hi^ transitional B cells and CD24^−^CD38^+^ plasmablasts, pre-gated on CD45^+^CD19^+^ B cells. Graphs displaying (**b**) frequencies of CD24^+^CD38^hi^ transitional B cells and CD24^−^CD38^+^ plasmablasts and (**c**) the mean fluorescence intensity (MFI) of CD5 on transitional B cells in HC (n = 20) and in patients with AN at T0 (n = 20) and at T1 (n = 20). (**d**) Representative dot plots of HC and patients with AN showing CD1d^+^CD5^+^ B cells, pre-gated on CD45^+^CD19^+^ B cells. (**e**) Graph displaying frequencies of CD1d^+^CD5^+^ B cells in HC (n = 20) and in patients with AN at T0 (n = 20) and after six weeks of multi-modal therapy at T1 (n = 20). *P-*values vs. HC were calculated by *t* test for independent samples, T0 vs. T1 by *t* test for paired samples, *p < 0.05; **p < 0.01; ***p < 0.001.

We next compared frequencies of immature transitional B cells and plasmablasts in adolescents with AN at T0 and T1 and in HC. For this, surface expression levels of CD24 and CD38 were assessed to differentiate CD24^+^CD38^hi^ transitional B cells from CD24^−^CD38^hi^ plasmablasts^28^. We found increased percentages of CD24^−^CD38^hi^ plasmablasts, a short-lived B cell subset capable of antibody secretion^20^, in AN at T1 when compared to HC (Fig. 4b). We next analyzed immature “transitional” B cells with earlier ascribed immunoregulatory capabilities that co-express high levels of CD24 and CD38^20^. We found markedly reduced CD24^+^CD38^hi^ transitional B cell frequencies at T0 and T1 when compared to HC (Fig. 4a,b). We also evaluated expression levels of CD5 on transitional B cells, an immunoreceptor associated with the human B cell receptor complex^29^, by measurement of mean fluorescence intensity (MFI). Interestingly, transitional B cells of AN patients expressed lower levels of CD5 at T0 when compared to transitional B cells in HC. CD5 surface expression maintained at lower levels after AN treatment (Fig. 4c).

We further studied CD1d^+^CD5^+^ B cells, another B cell subset with well-established immunoregulatory capacities^30^. AN patients at T0 exhibited markedly reduced frequencies of CD1d^+^CD5^+^ B cells in comparison to HC (Fig. 4d,e). At T1, CD1d^+^CD5^+^ B cell percentages were further reduced in AN when compared to T0 and HC. Altogether, our findings demonstrate that transitional B cells and CD1d^+^CD5^+^ B cells, both of which harbor immunosuppressive capacities, are reduced in PB of adolescents with AN.

### PhenoGraph analysis identifies alterations in CD19^+^ B cell subsets in AN

**Figure 5:**
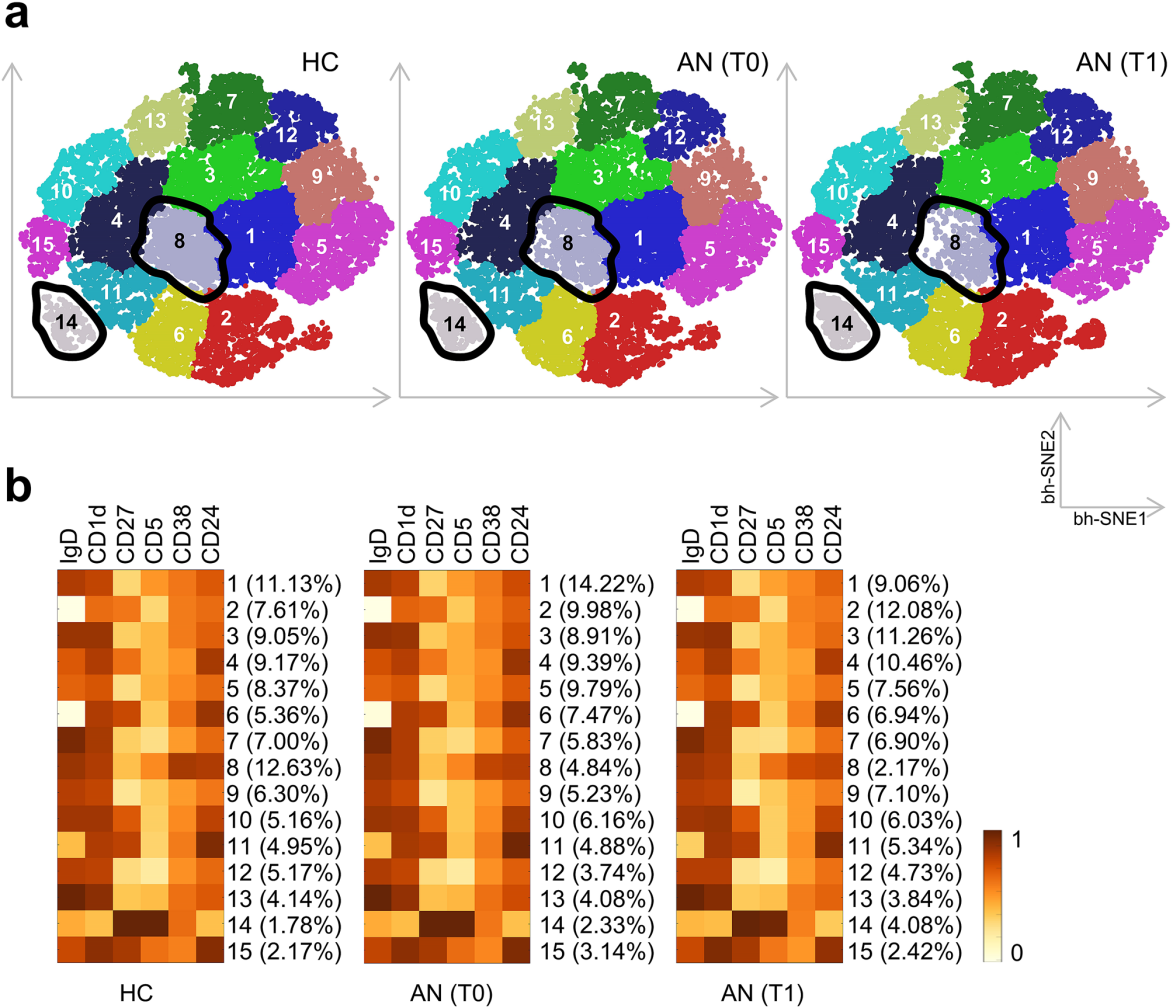
B cell markers in PBMC in patients with anorexia nervosa (AN) and healthy controls (HC). PBMC of HC and adolescents with AN at T0 and six weeks after multi-modal therapy (T1) were stained for CD45 and CD19 expression. (**a**) Each graph consists of 10,000 cells merged from the respective group of participants (HC n = 20; patients with AN at T0 n = 24; patients with AN at T1 n = 20). Cells were clustered based on CD45 and CD19 expression using the PhenoGraph algorithm, after using the Barnes-Hut Stochastic Neighbor Embedding (bh-SNE) algorithm to cluster cells with similar expression patterns of CD1d, CD5, CD24, CD27, CD38 and IgD. (**b**) Heatmaps showing the percentages of metaclusters (Y-axis) and mean expression levels of six molecules by individual metaclusters (X-axis) in the different conditions: HC, AN(T0), AN(T1). Cell clusters and heat maps were generated by using the Matlab software (http://www.mathworks.com/products/matlab/).

In addition to conventional flow cytometry data analysis, we utilized an unsupervised clustering approach to characterize B cell populations based on surface marker expression profiles in greater detail. Using PhenoGraph clustering analysis, we identified 15 populations of CD19^+^ B cells based on the distinct expression profile of IgD, CD1d, CD5, CD24, CD27, and/or CD38 (Fig. 5a). For data condensation and two-dimensional representation, we applied the Barnes-Hut stochastic neighbor embedding (bh-SNE) algorithm. By this clustering approach, we found mild alterations in surface marker expressions in the majority of B cell populations between HC, AN at T0, and AN at T1, with the exception of two B cell subpopulations. Specifically, B cell frequencies that co-express CD24, CD38, IgD, and CD1d (population 8), thus most likely comprising transitional B cells, were 2.6 times lower at T0 in AN (4.84%) when compared to the corresponding B cell subset in HC (12.63%). At T1, frequencies of these cells were further reduced by approximately 50% (2.17%) (Fig. 5b). We further determined a B cell subset that co-expressed CD27 and only low levels of IgD, thus potentially representing IgD^−^CD27^+^ switched memory B cells (population 14) in HC (1.78%). This B cell subset increased 1.3 times at T0 (2.33%) and more than twofold (4.08%) at T1 when compared to HC (Fig. 5b). Interestingly, clustering analysis further revealed, that this B cell subset co-expressed high levels of CD5. Conventional manual re-gating of our flow cytometry data confirmed co-expression of CD5 on IgD^−^CD27^+^ B cells with equivalent CD5 expression levels in AN and HC (data not shown). In sum, PhenoGraph clustering analysis validates our results yielded from conventional gating of flow cytometric data and points toward a reduction in transitional B cell and an increase in IgD^−^CD27^+^ B cell percentages in AN versus HC.

### Close link between specific B cell subsets and body composition parameters in AN

To examine relationships between B cell subsets and body composition parameters in AN and HC, we performed correlational studies (see Supplementary Table S2). In HC, frequencies of lymphocytes correlated inversely with the percentage of (FM) (ρ − 0.460; *p* < 0.05). In AN at T0, a positive correlation was found between frequencies of regulatory CD1d^+^CD5^+^ B cells and the percentage of FM (ρ 0.456; *p* < 0.05) and (FMI) (ρ 0.474; *p* < 0.05). Also, expression levels of CD5 on transitional B cells correlated positively with FM (ρ 0.438; *p* < 0.05) and FMI (ρ 0.428; *p* < 0.05). At T1, FM and FMI correlated positively with CD5 surface expression on transitional B cells (ρ 0.557, ρ 0.568; *p* < 0.05). At this time point, we further observed a positive correlation between regulatory CD1d^+^CD5^+^ B cells and BMI SDS (ρ 0.485; *p* > 0.05) and transitional B cell frequencies with FFMI in AN (ρ 0.477; *p* < 0.05).

Multivariate linear regression analyses (Table 2) revealed that a model which included age (β = 0.325, *p* < 0.05), transitional B cell frequency (β = 0.222, *p* = 0.133), CD5 MFI on transitional B cells (β = 0.832, *p* < 0.001), and CD1d^+^CD5^+^ B cell frequency (β = − 0.462, *p* < 0.05) contributed to the variance of BMI of 44 participants [HC = 20, AN(T0) = 24] and explained 34.8% of the variance [F (4,39) = 5.193, *p* < 0.01; R = 0.590, R^2^ = 0.348]. Our analysis demonstrates that age, expression levels of CD5 on transitional B cells, and the frequency of CD1d^+^CD5^+^ B cells are significant predictors of participants’ BMI. For FMI, a significant regression equation was found [F (4,37) = 5.127, *p* < 0.01] with an R of 0.601 and an R^2^ of 0.361 for 42 participants [HC = 19, AN(T0) = 23]. In this model, age (β = 0.344, *p* < 0.05) and CD5 MFI on transitional B cells (β = 0.613, *p* < 0.05) were identified as significant predictors for FMI. These analyses demonstrate a close link between body composition parameters and the immunoregulatory B cell compartment in patients with AN.

**Table 2:** Multivariate linear regression analyses of BMI and FMI in anorexia nervosa.

Variables	B	SE	Beta	t	*p*-value
**BMI**					
(Constant)	5.761	4.348		1.325	0.193
Age	0.615	0.269	0.325	2.288	**0.028**
Transitional B cells	0.037	0.024	0.222	1.534	0.133
CD5 MFI on transitional B cells	0.001	0.000	0.832	4.091	**0.000**
CD1d^+^CD5^+^ B cells	− 0.220	0.097	− 0.462	− 2.275	**0.028**
**FMI**					
(Constant)	− 4.180	2.601		− 1.607	0.116
Age	0.395	0.161	0.344	2.455	**0.019**
Transitional B cells	0.019	0.016	0.202	1.210	0.234
CD5 MFI on transitional B cells	0.001	0.000	0.613	2.360	**0.024**
CD1d^+^CD5^+^ B cells	− 0.039	0.079	− 0.130	− 0.490	0.627

Significant effects are in bold print.

*MFI* mean fluorescence intensity

Body mass index (BMI): F = 5.193, df (4,39), *p* = 0.002; R = 0.590, R^2^ = 0.348.

Fat mass index (FMI): F = 5.217, df (4,37), *p* = 0.002; R = 0.601, R^2^ = 0.361.

## Discussion

Several studies in AN demonstrated dysregulated humoral immune responses involving antibody production by B cells^21,31^. However, knowledge on the composition of PB B cell compartment in AN is limited and treatment effects and the association with body compositions have not been fully evaluated. In this study, we found a specific B cell profile in PB of patients associated with the acute AN stage at admission and a time point after 6 weeks of therapy. We further observed strong relationships between body composition parameters and the immunoregulatory B cell compartment.

In accordance with earlier studies, we found equivalent frequencies of overall B cells in adolescents with AN before therapy when compared to healthy individuals^17,21–23^. Regarding numbers and percentages of total lymphocytes, there is discrepancy in the literature, and relative lymphocytosis as well as lymphopenia in PB have been found in AN^17,32–36^. In our study, patients with AN displayed reduced percentages of total PB lymphocytes and B cells exclusively at T1 when compared to HC. It is well established that glucocorticoids may induce lymphocyte apoptosis and hypercortisolemia is frequently occurring in starved patients with AN^37^. However, it is unlikely that this scenario is causative for decreased proportions of B cells observed exclusively at T1, but not at inpatient admission. This seemingly paradoxical finding could be caused by alternative mechanisms: (i) a delayed reactivity of the immune system to starvation similar to that reported for metabolic parameters, (ii) changes in the intestinal microbiota due to refeeding, and (iii) stressors related to the inpatient setting, (e.g. psychological stress)^3,9^.

We further determined detailed immunophenotyping of circulating B cell subsets in AN using flow cytometry analysis of combinatorial surface marker sets. We observed no changes in frequencies of late Bm5 cells in PB at the acute stage of AN. However, percentages of these antigen-experienced B cells were increased in anorectic individuals at T1, namely after 6 weeks of multimodal treatment. In addition, antigen experienced IgD^−^CD27^+^ class-switched memory B cells increased at T0 compared to HC and further increased at T1. It is well established, that CD27^+^ memory B cells have undergone Ig class switching for rapid production of IgG, IgA or IgE antibodies in secondary immune responses. It has further been shown, that B cell class switching is controlled by cytokines such as IL-4 and IL-6^38^. Increased IL-6 blood levels in patients with AN may thus promote B cell class switch^16^. Strikingly, these antigen-experienced B cell changes occurred after 6 weeks of inpatient therapy, and not in the acute phase of starvation. The multimodal therapeutic concept offered in this study included nutritional counselling and treatment^39^. Under healthy conditions, the intestinal barrier forms a barrier against pathogenic microorganisms and oral tolerance mechanisms maintain a state of unresponsiveness to ingested dietary antigens^40^. However, an increased permeability for antigens of the colon has been demonstrated in an activity-based AN mouse model and in AN^41^. Resumption of dietary antigens by anorectic patients during therapy may therefore cause increased memory B cell percentages at T1.

Leakiness of gut food antigens and potential break down of oral tolerance may also partially explain the mild inflammatory state and enhanced risk of autoimmune diseases in AN. A fraction of patients with AN has also been shown earlier to display enhanced blood levels of autoantibodies against e.g. neuropeptide α-melanocyte-stimulating hormone (α-MSH) and adrenocorticotropic hormone (ACTH), the latter involved in the stress response of the hypothalamic–pituitary–adrenal (HPA) axis^42^. Also higher levels of IgG, IgM, and IgA autoantibodies against ghrelin, a peptide hormone that stimulates food intake, have been found in anorectic individuals^43^. Future studies are therefore required for measurement of serum IgG, IgA and IgM antibody activities against food antigens in AN. On the other hand, we found reduced frequencies of antigen inexperienced naïve IgD^+^CD27^−^ B cells in patients with AN. A murine study of fasting/refeeding demonstrated that fasting induced migration of naïve B cells from Peyer’s patches (PP) to bone marrow (BM), while refeeding triggered naïve B cell trafficking from BM to PP in response to chemokine gradients^44^. In analogy, reduced frequencies of naïve B cells in anorectic adolescents may be caused by altered compartmentalization of naïve B cells in PB due to restricted diet and “refeeding”.

A prominent finding of our study is that frequencies of CD1d^+^CD5^+^ B cells and transitional CD24^+^CD38^hi^ B cells, both shown to harbor immunoregulatory capacities^28,30^, were markedly reduced in AN patients. While the latter regulatory B cell subset maintained at low levels, CD1d^+^CD5^+^ B cells even further decreased at T1. Regulatory B cells (B_reg_) fulfill immunomodulatory functions and maintain self-tolerance, via e.g. production of anti-inflammatory cytokines. Accordingly, decreased numbers and impaired functions of B_reg_ cells have been found in autoimmune diseases such as multiple sclerosis, systemic lupus erythematosus, and rheumatoid arthritis^25^. Reduced percentages of phenotypically regulatory B cells in AN point toward an impaired function of the B_reg_ cell compartment further contributing to an enhanced risk of developing autoimmunity in AN. Also plasmablasts, besides their antibody production capacity, may possess immunoregulatory capacities^45^. The observed increase in circulating plasmablasts in AN may therefore substitute lower percentages of CD1d^+^CD5^+^ and transitional B cells. In contrast to the observed changes in the B_reg_ compartment in AN, earlier studies demonstrated that regulatory T (T_reg_) cell proportions were not affected in AN^9^.

A further noteworthy finding of our study was that expression of CD5 on transitional B cells is downregulated in patients with AN when compared to HC. CD5 is a scavenger-like receptor that recognizes pathogen-associated molecular patterns (PAMP) on fungal surfaces. CD5 further inhibits B cell receptor signaling for maintenance of self-tolerance and is implicated in survival of human and mouse B cells^29^. It is therefore possible, that low levels of CD5 on transitional B cells and CD1^+^CD5^+^ B cells affect PAMP recognition and B cell survival in AN. In healthy individuals, downregulation and intracellular accumulation of CD5 in B cells has been reported upon exposure to IL-6^46^. Thus, increased IL-6 levels found in AN^16^ may cause downregulation of CD5 expression on transitional B cells. In accordance with our findings, a recent study determined lower CD5 plasma levels that correlated with BMI in anorectic patients^47^. Our additional regression analyses revealed that expression levels of CD5 on transitional B cells may serve as a predictor for BMI and FMI in patients with AN. Furthermore, CD1d^+^CD5^+^ B cell frequencies contributed to the variance of BMI.

Notably, our findings demonstrate that regulatory CD1d^+^CD5^+^ B cell percentages positively correlate with FMI at the acute stage of starvation, emphasizing the strong relationship between the regulatory B cell compartment and severity of disease. Another study reported associations between B cell counts and BMI SDS in anorectic adolescents^17^. We demonstrated that FM and FMI, but not BMI parameters, correlated with the B cell phenotype at T0 and T1. Accordingly, adipose tissue might specifically impact B cell mediated immune regulation at the acute stage of starvation and during weight/fat gain. The hormone leptin is a regulator of adipose tissue metabolism and may play an essential role in these processes^48^. Leptin blood levels have been shown to inversely correlate with BMI and FM in patients with AN. Therapy-induced weight gain instead resulted in hyperleptinemia^49^. Interestingly, leptin also exerts proinflammatory activity and promoted lymphopoiesis in mice causing a rise in immature BM B cells^50,51^. It is thus possible, that here found alterations of immature B cell percentages in AN depend on leptin blood levels. Just like starvation, realimentation thus alters metabolic^3^ and immune profiles and may critically impact the development and course of AN^52^. Future studies are needed to determine the diagnostic and prognostic impact of immunological parameters in the context of metabolomic parameters during the acute phase and recovery of AN.

A limitation of our study is the small albeit homogenous cohort thus precluding generalized conclusions. We considered 6 weeks of inpatient induced realimentation as sufficient to assess pre-post parameters; obviously, additional time points including time points such as attainment of target weight would allow a better interpretation of the differences between T0 and T1 observed in this study. Furthermore, functional studies are required to clarify the effects of AN on functionally diverse B cell subsets. In addition, larger cohorts have to be enrolled in future studies and a follow-up study may unravel the long term treatment effects on phenotype and function of B cell subsets in AN and fully remitted individuals. An open question is still the clinical significance of herein described B cell signatures for disease predisposition and immune defense in anorectic individuals. Exposure to infection and hospital-treatment of infections in childhood has been associated with an enhanced risk of ED in adolescent girls^53^. Future studies have to unravel whether childhood infections induce long term alterations of the regulatory B cell compartment and therefore contribute to the risk of developing AN. Taken together, our results demonstrate an altered B cell compartment in PB in AN, characterized by decreased frequencies of regulatory B cells and the maintenance of a dysregulated B cell compartment after 6 weeks of multi-modal therapy. Overall, our study underscores that the nutritional status alters the B cell phenotype that is associated with body composition parameters in adolescents with AN.

## Methods

### Subjects

Patients with AN (n = 24) and healthy controls (HC, n = 20) were recruited at the Department of Child and Adolescent Psychiatry, Psychotherapy, and Psychosomatics, University Hospital Essen, University of Duisburg-Essen, Essen, Germany. This study was performed in accordance with the declaration of Helsinki and approved by the ethics committee of the Medical Faculty of the University of Duisburg-Essen (12-5289-BO). All patients and controls and their parents gave written informed consent prior to the study. All patients with AN were admitted to psychiatric inpatient care. They were treated according to the German S3-guidelines^54^ with a multimodal, cognitive and dialectical behavioral therapeutic concept^39^. The treatment concept has been individually composed of the following modules: individual and group psychotherapy (skills groups and individual skills trainings), creative- and physical therapy, family therapy, regular medical visits, nutritional counselling/psychoeducation by specially trained nursing staff, eating assistance, as well as shopping and cooking groups, social work counseling and group care services. A weight gain of at least 500 g per week and a minimum target weight in the range of the 20–25th age-adjusted BMI percentile were aimed for^39^.

Inclusion criteria for the group of HC were female gender, German ancestry and age 12–18 years. Inclusion criteria for the AN group were a diagnosis of AN, female sex, German ancestry and age from 12 to 18 years. The diagnosis of AN was confirmed via clinical examination and a semi structured interview: the Kiddie Schedule for Affective Disorders and Schizophrenia (K-SADS) according to the Diagnostic and Statistical Manual of Mental Disorders, fourth edition, text revision (DSM-IV-TR)^55^. BMI of one patient was on the 12th age- and sex adjusted BMI percentile; but as all other DSM-IV-TR criteria for AN were fulfilled the patient was included in our study. Exclusion criteria for patients and HC were a severe comorbid mental disorder, alcohol or drug abuse, chronic endocrinological or inflammatory diseases, cancer, insufficient German language skills and IQ < 70. Psychiatric disorders and severe somatic diseases were excluded based mainly on participants’ medical and psychiatric history and by application of Strengths and Difficulties Questionnaire (SDQ), a brief behavioral screening questionnaire for 3–16 year olds. The SDQ covers 25 items which are divided into 5 sub-classes: emotional symptoms, conduct problems, hyperactivity/inattention, peer relationship problems and prosocial behavior^56^. Anthropomorphic measurements were done at the acute stage of starvation (T0) and 6 weeks after admission. Body weight and height at referral were measured according to a standard operating procedure (SOP) using the same calibrated scale and stadiometer for all study participants (Seca). All adolescents were weighed in underwear without shoes. Information on body composition including fat free mass (FFM, in kg and %) and fat mass (FM, in kg and %) were derived from air displacement plethysmography using the thoracic gas volume method BodPod^57^ and software V4.2 + as supplied by the manufacturer (Concord, CA: Life Measurement Inc.). BodPod measurements were conducted in underwear with metal objects (e.g. watches) removed. Physical activity was not allowed two hours prior to measurements. Fat mass index (FMI) and fat free mass index (FFMI) were calculated dividing FM and FFM by the square of body height (kg/m^2^), respectively. BMI was calculated by dividing weight from the BodPod data by the square of height (kg/m^2^). On the basis of German reference data for children^58^, individual BMI values were transformed into BMI standard deviation scores (BMI SDS) and BMI centiles using the method suggested by Cole^59^. BMI SDS approximates the deviation of an individual BMI from the median of the reference group expressed in units of the standard deviation. The calculation of premorbid BMI was based on the parents’ recalled premorbid weight divided by the square of measured height at inpatient admission according to Coners et al*.*^60^.

All participants were non-smokers (n = 44). All individuals in the control group showed a regular menstrual cycle (n = 20) whereas 21 anorexic patients were amenorrheic. Three patients with AN took oral contraceptives and reported a withdrawal bleeding. One patient with AN received Benzodiazepine and Domperidone at T0. Three patients with AN took Vitamin D due to Vitamin D deficiency and three patients took oral contraceptives at T0. None of the subjects in the control group took medication (see Supplementary Table 3). Regression analyses revealed that supplementation of Vitamin D had no effect on B cell parameters. Since only 1 individual was on psychopharmacological treatment an additional statistical analysis was not feasible.

### Blood sample collection

Blood was obtained in the morning after an overnight fast at the acute stage of starvation (T0) within the first week of hospitalization and after 6 weeks of inpatient treatment (T1). 4 of 24 patients with AN rejected their consent for the follow up blood donation. 15 ml freshly drawn peripheral venous blood from the participants was collected in sterile sodium heparin-treated tubes. To separate peripheral blood mononuclear cells (PBMC), a standard density gradient centrifugation using Leucosep tubes (Greiner Bio-One) was performed according to the instructions of the manufacturer. Briefly, 50 ml Leucosep tubes were preloaded with 15 ml Ficoll-Paque PLUS by centrifugation for 30 s at 1000×*g*. Next, 30 ml of whole blood and PBS (1:1) was added to the Leucosep tube. After centrifugation for 10 min at 1000×*g* without brakes at room temperature (RT), the cell suspension was collected. Cells were washed three times with PBS supplemented with 2% fetal calf serum (FCS) for 10 min at 300×*g*, respectively. Before counting, cells were resuspended in X-Vivo 15 (Lonza) supplemented with 10% FCS.

### Cryopreservation and thawing of PBMC

Cryopreserved aliquots of purified PBMC were stored in the gas phase of liquid nitrogen. Therefore, 900 µl X-Vivo 15 supplemented with 10% FCS containing 1 × 10^7^ cells were mixed with 900 µl freezing medium consisting of 80% FCS and 20% dimethyl sulfoxide (DMSO). The cryovials were immediately stored at − 80 °C overnight and later transferred to the liquid nitrogen container. On the day of use, cells were slightly thawed in a 37 °C water bath. In a next step, the cell suspension was transferred into a 15-ml tube prefilled with 10 ml PBS. Following centrifugation for 7 min at 330×*g*, cells were washed once with PBS supplemented with 2% FCS.

### Flow cytometry

An 8-color panel for immunophenotyping was designed to identify B cell subsets of diverse maturation stages among PBMC according to our earlier publication in whole blood^61^. In brief, PBMC were stained with fluorochrome-conjugated antibodies (see Supplementary Table S4) at RT protected from light for 30 min. Cells were washed once with PBS supplemented with 2% FCS and finally resuspended in staining buffer (PBS supplemented with 2% FCS and 0.1% NaN_3_). Samples were acquired on a CytoflexS flow cytometer (Krefeld, GER: Beckman Coulter GmbH) and analyzed by FlowJo Software v10 (Ashland, OR: Becton, Dickinson and Company; 2019). All antibodies were purchased from BioLegend (San Diego, CA). Exemplified gating strategies (see Supplementary Fig. S2) and fluorescence minus one (FMO) controls (see Supplementary Fig. S3) are shown in supplementary material.

### Unsupervised cluster-analysis of high-dimensional flow cytometry data

The unsupervised visualization tool programme viSNE for MATLAB version 2019b (Natick, MA: The MathWorks Inc.) was used to analyse the 8-colour flow cytometry data as published before^62^. Briefly, the Barnes-Hut Stochastic Neighbour Embedding (bh-SNE) algorithm was applied for data condensation and cell clustering^63,64^. Prior to clustering, data was additionally normalized^65^. Final visualization of cell clusters and heat maps was performed using the PhenoGraph method^66^ in MATLAB (http://www.mathworks.com/products/matlab/).

### Statistical analysis

Data were evaluated using IBM SPSS version 25 (Armonk, NY: IBM Corp) and visualized with GraphPad Prism version 5 (San Diego, CA: Graphpad). Shapiro–Wilk test was used to test continuous sample data for normal distribution. Differences between patient subgroups for normally distributed data (according to the Kolmogorov–Smirnov test) were evaluated using the two-tailed *t* test for paired (T0 vs. T1) or independent (HC vs. T0; HC vs. T1) samples. For non-normally distributed data, differences were assessed using the Wilcoxon Signed-Rank test or Mann–Whitney *U* test, respectively. For correlational studies, Kolmogorov–Smirnov test for normal distribution was used. Subsequently, either Pearson or Spearman correlation coefficient was used, as appropriate. Furthermore, the influence of age and parameters of phenotypically regulatory B cell subsets (transitional B cells, CD1d^+^CD5^+^ B cells and CD5 MFI on transitional B cells) on the variance of BMI and FMI was calculated by multivariate linear regression. *p < 0.05; **p < 0.01; ***p < 0.001 were considered statistical significant.

## Supplementary Information

Supplementary Information.

## Data Availability

The datasets generated during and/or analyzed during the current study are available from the corresponding author on reasonable request.
