# Supplementary material for: Alterations in B cell subsets correlate with body composition parameters in female adolescents with anorexia nervosa
Source: Sci Rep. 2021 Jan 13;11:1125. doi: 10.1038/s41598-020-80693-4 (PMC7806719; doi:10.1038/s41598-020-80693-4)
Supplement: Supplementary file 1 — Supplementary Information. [file 41598_2020_80693_MOESM1_ESM.pdf]

Supplementary Information

Alterations in B Cell Subsets Correlate with Body Composition Parameters in Female Adolescents with Anorexia Nervosa

Jana Freff [1,2], Kathrin Schwarte [1], Lisa Bröker [1,2], Judith Bühlmeier [3], Isabelle Kraft [3], Dana Öztürk [3], Anke Hinney [3], Volker Arolt [1], Udo Dannlowski [1], Georg Romer [4], Bernhard T. Baune [1,5,6], Johannes Hebebrand [3], Manuel Föcker [3,4*], Judith Alferink [1,2*]

1 Department of Mental Health, University of Münster, 48149 Münster, Germany

2 Cells in Motion Interfaculty Cluster, University of Münster, 48149 Münster, Germany

3 Department of Child and Adolescent Psychiatry and Psychotherapy, University Hospital Essen, University of Duisburg-Essen, 45147 Essen, Germany

4 Department of Child and Adolescent Psychiatry and Psychotherapy, University Hospital Münster, 48149 Münster, Germany

5 The Florey Institute of Neuroscience and Mental Health, The University of Melbourne, 3010 Melbourne, Australia

4 Department of Psychiatry, The University of Melbourne, 3010 Melbourne, Australia

* equal last authorship

Corresponding author:

Judith Alferink, Judith.Alferink@ukmuenster.de; University of Münster, Department of Mental Health, Albert-Schweitzer-Campus 1, 48149 Münster, Germany

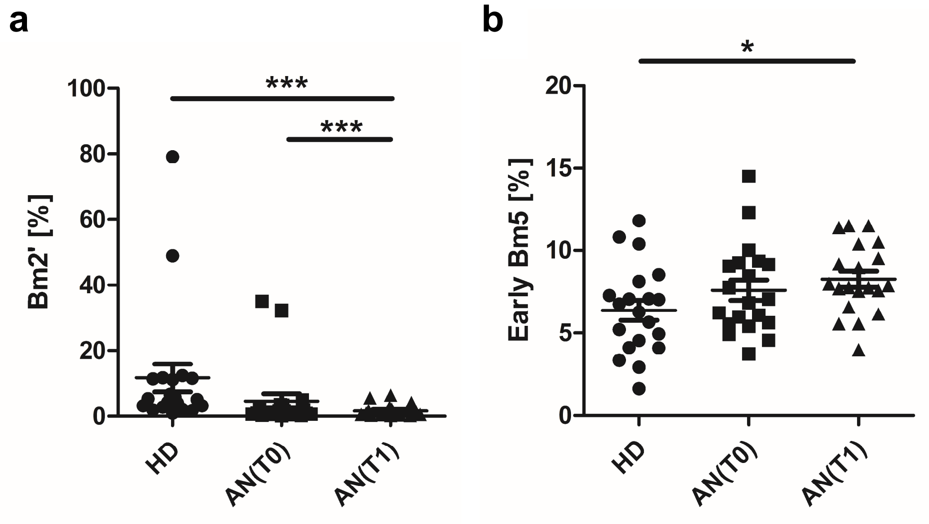

Figure S1. Mature B cell (Bm) subpopulations in PBMC of healthy controls (HC) and adolescents with anorexia nervosa (AN). Graphs displaying frequencies of (a) Bm2' cells and (b) early Bm5 cells in HC (n=20) and AN at T0 (n=20) and at T1 (n=20). *P*-values vs. HC were calculated by *t* test for independent samples, T0 vs. T1 by *t* test for paired samples, *$p<0.05$; ***$p<0.001$.

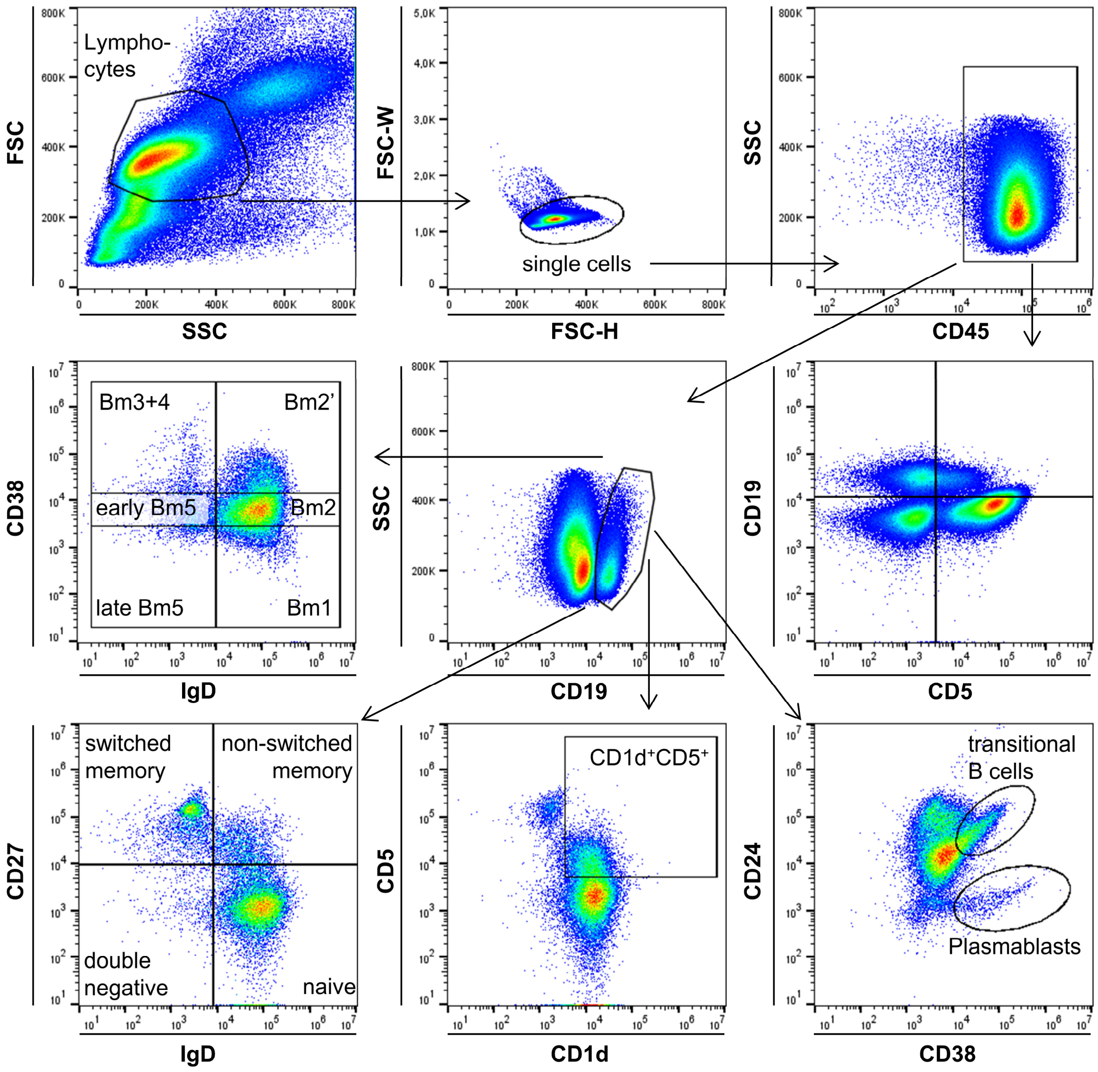

Figure S2. Exemplary gating strategy for phenotypic characterization of B cell subsets in adolescents with anorexia nervosa (AN) and healthy controls (HC). Lymphocytes from peripheral blood mononuclear cells (PBMC) were selected from total events based on their size (forward scatter [FSC]) and granularity (side scatter [SSC]). Single cells were gated based on FSC-Width vs FSC-Height information. Pre-gated on $CD45^+$ and $CD19^+$, cells were further gated for transitional B cells ($CD24^{high}CD38^+$) and plasmablasts ($CD24^{low}CD38^+$), for $CD1d^+CD5^+$ regulatory B cells, as well as for naïve (IgD^+CD27^-), switched memory (IgD^- $CD27^+$), and non-switched memory B cells (IgD^+CD27^+). Additionally, B cells were subdivided into Bm1, Bm2, Bm2', Bm3+4 and early and late Bm5 cells based on CD38 vs. IgD expression.

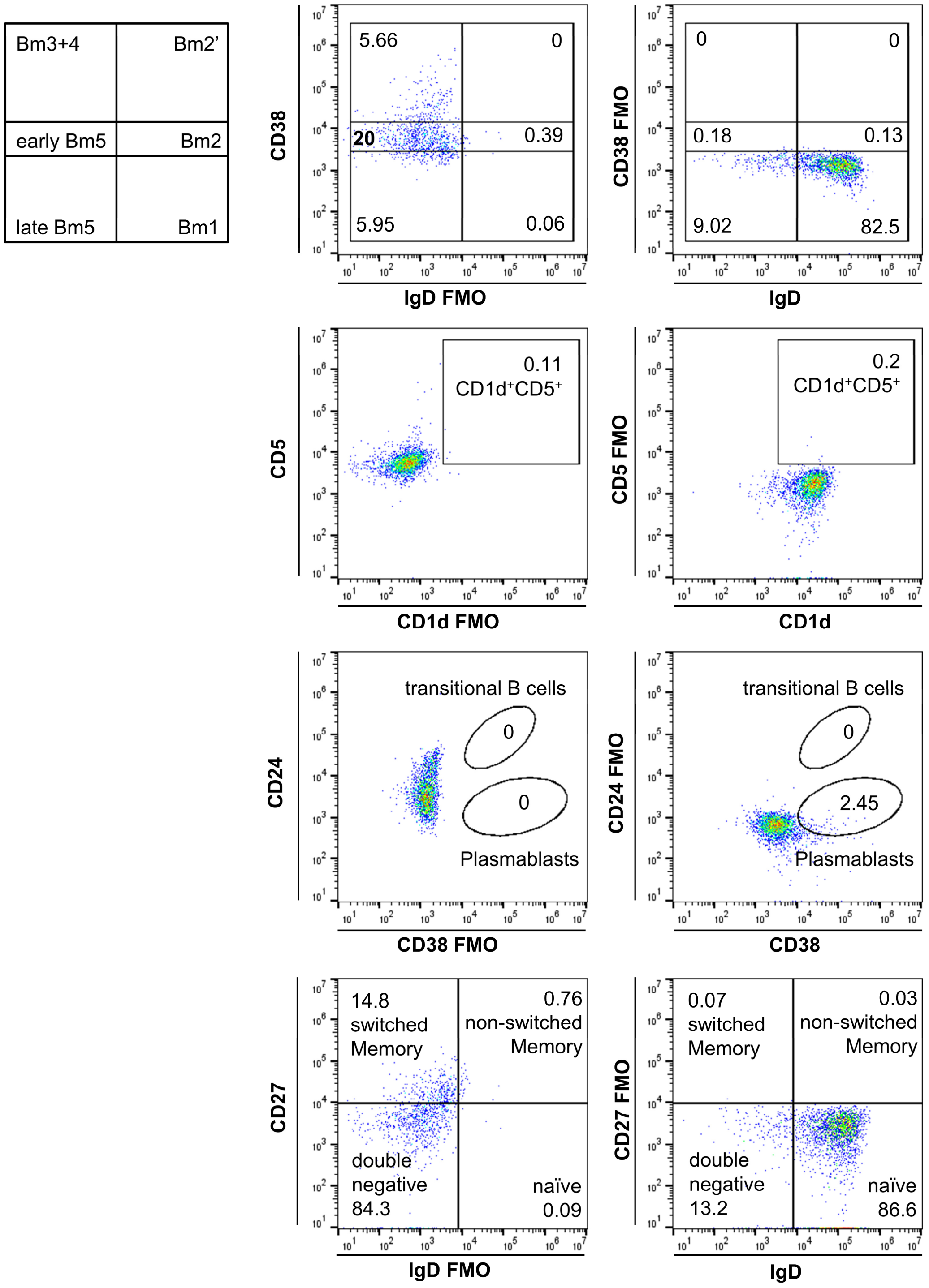

Figure S3. Representative fluorescence minus one (FMO) controls for the corresponding gating strategy.

Table S1. Clinical characteristics of patients with anorexia nervosa (AN) at T0.

	**AN (T0) (n=24)**
Premorbid BMI (kg/m^2, mean ± SD)	20.4 ± 2.8$^+$
Premorbid BMI percentile (mean ± SD)	45.2 ± 28.3$^+$
Premorbid BMI SDS (mean ± SD)	-0.1 ± 0.9$^+$
Inpatient pretreatment (yes/no, n)	5/19

$^+$n=23; BMI: body mass index; SDS: standard deviation score.

Table S2. Correlation coefficients of immune parameters with clinical characteristics for HC, AN(T0) or AN(T1).

	HC (n=20)						AN(T0), (n=24)						AN(T1), (n=20)					
	BMI SDS (KIGGS)	BMI percentile	BMI [kg/m^2]	FM [%] (n=19)	FMI [kg/m^2] (n=19)	FFMI [kg/m^2] (n=19)	BMI SDS (KIGGS)	BMI percentile	BMI [kg/m^2]	FM [%] (n=23)	FMI [kg/m^2] (n=23)	FFMI [kg/m^2] (n=23)	BMI SDS (KIGGS)	BMI percentile	BMI [kg/m^2]	FM [%]	FMI [kg/m^2]	FFMI [kg/m^2]
Lymphocytes	.121	.135	-.065	**-.460***	-.396	.325	*-.268*	*-.268*	-.239	*-.001*	.061	-.276	*.046*	-.093	.148	.052	.008	.082
CD19$^+$ B cells	-.218	-.262	-.107	.102	.048	-.211	*.122*	*.122*	*.123*	*.017*	*.057*	*.296*	*.362*	*.362*	*.163*	*.159*	*.178*	*.017*
Naive B cells	-.037	.032	-.101	.301	.219	-.405	*.274*	*.274*	.164	*.105*	.175	-.087	*.211*	.285	.067	.183	.134	-.080
Non-switched memory B cells	-.002	-.067	.128	-.144	-.095	.288	*-.297*	*-.297*	-.347	*.223*	.072	-.311	*-.218*	-.136	-.001	.299	.312	-.240
Switched memory B cells	.113	.036	.147	-.244	-.154	.402	*-.367*	*-.367*	-.125	*-.197*	-.172	.099	*-.268*	-.283	-.055	-.174	-.163	.079
Plasmablasts	*.245*	*.245*	*.338*	*.225*	*.405*	*.180*	*-.029*	*-.029*	*-.161*	*-.208*	*-.247*	*-.202*	*-.120*	*-.120*	*-.220*	*.047*	*.120*	*-.099*
Transitional B cells	*-.262*	*-.262*	*-.087*	*.047*	*.093*	*-.211*	*.119*	*.119*	*.050*	*.230*	*.205*	*-.100*	*.435*	*.435*	*.305*	*-.012*	*.021*	***.477****
Late Bm5 cells	.173	.198	.118	-.121	-.026	.226	*-.175*	*-.175*	.041	*.066*	.128	.181	*-.340*	-.444	-.112	.143	.144	-.194
CD1d$^+$CD5$^+$ B cells	.037	.067	.082	.171	.155	-.061	*.213*	*.213*	-.087	***.456****	**.474***	-.003	***.485****	.421	.178	.253	.288	-.052
CD5 MFI on transitional B cells	.200	.243	.097	.032	.081	.056	*.190*	*.190*	.165	***.438****	**.428***	-.052	*.405*	.168	.159	**.557***	**.568****	-.303

$^{\#}$n=19, $^{+}$n=23; HC: healthy control: AN: Anorexia nervosa; BMI: body mass index; SDS: standard deviation score; KIGGS: German Health Interview and Examination Survey for Children and Adolescents; FM: fat mass; FMI: fat mass index; FFMI: fat free mass index; italics show Spearman Rho correlation coefficients, otherwise correlation is given according to Pearson; bold printed values mark significant correlations; *p <.05; **p<.01.

Table S3. Contraceptive, supplements and medication of patients with AN at T0 and T1.

	AN(T0)	AN(T1)
**Oral contraceptive**	3	3
**Supplements**		
Vitamin D	3	9
Nutritional drink (Fresubin)	2	2
Sodium glycerophosphate	1	0
Magnesium	0	2
**Benzodiazepine**	1	0
**Phenothiazine** (Promethazine)	0	1
**Domperidone**	1	1
**SSRI**	0	1
**Antipsychotic drug** (Quetiapine, low dose)	0	2
**Antibiotics** (Flucloxacillin)	0	1

Table S4. Antibodies used for extracellular staining.

Specificity	Fluorochrome	Clone
CD1d	PE	51.1
CD5	APC	UCHT2
CD19	PerCP	HIB19
CD24	BV510	ML5
CD27	PE-Cy7	M-T271
CD38	BV421	HIT2
CD45	APC-Cy7	HI30
IgD	FITC	IA6-2
